# Falling for a Diagnosis: West Nile Myelitis without Encephalitis

**DOI:** 10.7759/cureus.5838

**Published:** 2019-10-04

**Authors:** Vincent Chan, Artem Minalyan, Patrick Ottman, Ahmad Raza, Anubha Tewary

**Affiliations:** 1 Internal Medicine, Abington Hospital-Jefferson Health, Abington, USA

**Keywords:** west nile virus encephalitis, viral fever, west nile virus

## Abstract

Poliovirus has been eradicated in the US for 40 years. Its sequelae, poliomyelitis, a syndrome characterized by fever, meningitis, and flaccid paralysis, is a rare entity. Other viruses have been implicated in poliomyelitis-like syndromes since the elimination of poliovirus. West Nile virus (WNV), since its westward migration in 1999, has recently been found to be a causative agent of fever, encephalitis, and acute flaccid paralysis. We present the case of a male who presented to the hospital for fever and experienced a subsequent fall, without any symptoms of encephalitis, diagnosed with WNV infection.

## Introduction

Poliovirus, belonging to the enterovirus genus, has been eradicated in the US for 40 years. Its sequelae, poliomyelitis, is a syndrome characterized by fever, meningitis, and flaccid paralysis and is a rare entity in the Western world [1]. Poliomyelitis-like syndromes have been documented in a considerable manner in the Western world in recent years. West Nile virus (WNV), belonging to the flavivirus genus, has recently been found to be a rare agent of fever, encephalitis, and acute flaccid paralysis, all symptoms that were associated with poliomyelitis. Acute neurological disorders due to the aforementioned viruses are important in a clinician’s differential diagnosis of acute flaccid paralysis as serological confirmation can aid in early diagnosis and symptomatic management of its sequelae. While central nervous involvement presenting with meningitis and encephalitis is often found with these diseases [2], it is important to recognize that a minority of patients can also present without confusion or altered mental status. Management of the disease process is unclear and observation of complications that may arise, such as respiratory failure, is pertinent in the care of these patients. Prognostically, the outcomes are not always favorable for those who present with acute neurological disease. In this study, we discuss the case of a male who presented to the hospital with fever and acute flaccid paralysis with a subsequent brief review on the differential diagnosis of fever and acute flaccid paralysis of a viral origin.

## Case presentation

A 49-year-old male from Pennsylvania presented with complaints of fever and lethargy. One week prior to the presentation, he and his spouse had been traveling in the Pocono Mountains. A day into his travel, he had started to experience increased fatigue and a rise in temperature. This episode had seemingly self-resolved over the course of the day, and the patient had been back to normal the next day. However, he had continued to have intermittent symptoms of lethargy, fevers, and night sweats and had undergone a whole-day episode of loose stools even after his return from the mountains. Because of dysuria and urinary frequency, he had been seen at an urgent-care facility with an unremarkable urinalysis, complete blood count (CBC), and comprehensive metabolic panel (CMP). Subsequently, he had started to develop a fine petechial rash, most noticeable on his chest and upper extremities. He continued to be febrile with increasing body aches, episodes of emesis, and inability to tolerate oral intake. For these symptoms, he presented to the emergency department (ED) for continued care. 

In the ED, the patient was alert and oriented. He had a fever of 101.8 F, blood pressure of 122/63 mmHg, heart rate of 98 beats per minute, respiratory rate of 20 breaths per minute, and oxygen saturation of 97% on room air. The physical exam was unremarkable. He had no complaints of neck rigidity nor any confusion suggestive of meningitis or encephalitis. His blood work and imaging including CBC, CMP, hepatitis panel, and blood parasite smear were unremarkable. He had a mild lactic acidosis of 2.3 mmol/L. A chest X-ray was also unremarkable for any significant disease. He was admitted to the hospital with the diagnosis of sepsis and was administered broad-spectrum antibiotics.

Because of the patient’s recent travel history, rash, and fever, he was started on empiric doxycycline therapy for presumed arthropod-related illness. Serology for tick-borne illness and mosquito-borne illness were performed. On the evening of the day of admission, the patient was noticed by the nurse to be retaining urine, without an urge to urinate. He was bladder-scanned for 1,000 cc of urine and was catheterized, eventually relieving 1,100 ml of urine. Overnight, the patient’s fever rose to a maximum of 104.2 F. Because he was unable to sleep, the patient attempted to get out of bed and subsequently suffered a fall. This was described as the result of a sudden loss of strength in his lower extremities. When re-evaluated in the morning, the patient appeared significantly weaker, though he continued to be alert and oriented. In light of the fall, he was immediately evaluated by a neurologist. The patient’s neurological exam revealed him being alert and oriented, upper extremities 5/5 in strength, and lower extremities 2/5 on the right and 3/5 on the left in strength. No sensory deficits were observed. Lower-extremity reflexes were hypoactive bilaterally with negative Babinski sign bilaterally. His strength exam was normal on admission. A lumbar puncture was immediately performed, and the patient was subsequently taken for an MRI of his thoracic and lumbar spine.

**Figure 1:**
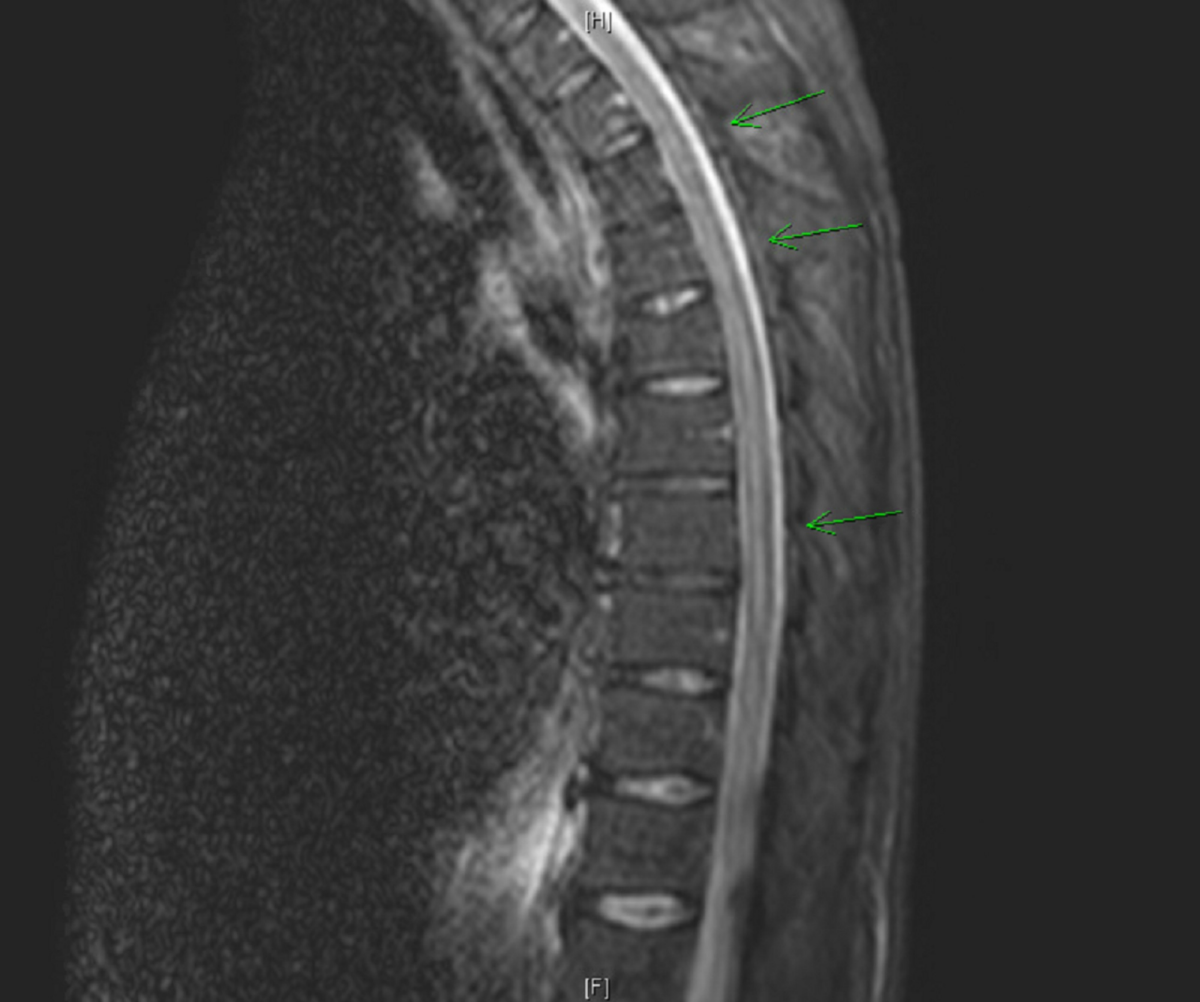
MRI demonstrating an abnormally increased intensity of the thoracic cord (green arrows)

The patient’s MRI demonstrated a subtle increased intensity of the distal thoracic cord with findings suggestive of enhancement of the traversing nerve roots (figure 1). The noted differentials at the time included transverse myelitis or a demyelinating process such as Guillain Barre syndrome (GBS). The patient was treated with pulse-dose steroids for presumed myelitis. 

The patient’s lumbar puncture studies demonstrated pertinent findings of a white blood cell (WBC) count of 592, lymphocytes of 83%, neutrophils of 15%, monocytes of 2%, red blood cell (RBC) count of 0, and no sign of xanthochromia. cerebrospinal fluid (CSF) protein was 163 mg/dL and glucose level was 56 mg/dL. The data were consistent with viral meningitis with pending viral serology. 

On the third day of admission, the patient was observed to have persistent urinary difficulty, and a urinary catheter was placed, which subsequently drained 1,000 cc of yellow urine. Initial Lyme serology returned as IgM and IgG positive, with a negative western blot confirmation. Between the fourth and sixth day of admission, tests for syphilis, Epstein-Barr virus, babesia, typhus, and rickettsia returned negative. Similarly, CSF polymerase chain reaction (PCR) was negative for enterovirus, herpes virus, and Lyme. Culture data to date including blood, CSF, and urine were negative for significant growth. WNV IgM serology returned positive during this period, with a negative IgG. CSF also confirmed an IgM positivity with IgG negativity. The patient was subsequently diagnosed with West Nile myelitis based on clinical presentation, CSF, and serology. 

The patient’s clinical exam was spread over the course of his stay in the hospital, with him eventually regaining most of his strength. His lower-extremity reflexes normalized, and his strength reached a peak of 3/5 on the right and 4/5 on the left at the time of his transfer to acute rehab with our physical medicine and rehabilitation team. 

## Discussion

Poliovirus is a single-stranded positive-sense RNA virus within the enterovirus genus. This virus was responsible for the poliomyelitis epidemic in the 1800s and 1900s. It is a virus transmitted via the fecal-oral route. Poliomyelitis, known in the literature as “polio”, is a disease characterized by fever, meningitis, and acute flaccid paralysis. Paralysis is the most feared complication of the triad and is seen in about 0.1-0.5% of infections [1]. This occurs when the virus enters the central nervous system and specifically replicates within the spine or brain. It specifically involves the anterior horn cells of the spinal cord, which results in isolated temporary or permanent motor paralysis without a sensory deficit. Diagnosis is often based on clinical exam alongside the testing of CSF. Prior to the discovery of the polio vaccination, these epidemics resulted in 13,000 to 20,000 cases of paralysis due to poliovirus in the US [2]. Since the discovery and implementation of vaccination against poliovirus, infections and poliomyelitis due to poliovirus have been thought to be eradicated in the US with no new cases reported in the country since 1979. 

While poliomyelitis is the condition most commonly associated with the complications caused by the enterovirus poliovirus, flaccid paralysis can also be caused by other viruses in the enterovirus genus, such as West Nile virus (WNV), herpes zoster virus, or rabies virus [3-6]. Poliovirus, a virus that has mostly been eradicated with vaccination, is still found in Asia and Africa and should be considered in the diagnosis of patients who have traveled to these areas [3]. Enteroviruses are usually associated with aseptic meningitis and encephalitis, but coxsackieviruses and echoviruses have also been linked to frequently reversible flaccid paralysis. Enterovirus type 71 paralysis has been documented in California [4,5]. Herpes zoster virus has also been shown to rarely cause motor paralysis outside of the facial muscles in Ramsey Hunt syndrome and can involve widespread myotomes compared to the dermatome-affected [6]. Finally, rabies virus can sometimes present as ascending paralysis from demyelination of the peripheral nerves and involvement of the central nervous system [7].

WNV belongs to the flavivirus genus and was first isolated in Uganda in 1937 [8]. While the virus initially was only found in the Middle East, Africa, Europe, Asia, and Australia, it spread to the Americas in 1999 through New York and is now found from Canada to Venezuela [9]. The virus is usually spread to humans and animals through infected mosquitos feeding on infected birds. Some human-human transmissions have occurred through organ transplants, blood transfusions, and breast milk [8]. Up to 80% of patients who contract WNV can be asymptotic. A minority of patients develop West Nile fever, which is accompanied by fever, fatigue, body aches, nausea, vomiting, rash on the trunk, and lymphadenopathy. An even rarer presentation results in neuroinvasive diseases. This devastating complication can include encephalitis, meningitis, and/or West Nile myelitis with symptoms consisting of high fever, headache, neck stiffness, stupor, disorientation, coma, tremors, convulsions, muscle weakness, bowel and bladder disruptions, and flaccid paralysis [10]. While neuroinvasive diseases can occur in anyone, those who are immunocompromised or those over the age of 50 are at the highest risk. The diagnosis of neuroinvasive diseases requires the detection of antibody titers in CSF [11].

Up to 75% of cases of WNV myelitis present with encephalitis, which was not present in our patient [12]. Pathogenically, it presents similarly to poliomyelitis. WNV myelitis uniquely presents with asymmetric areflexic or hyporeflexic weakness with a preserved sensory response. These findings are consistent with anterior horn demyelination, and WNV almost exclusively involves the anterior horn. Thus, these findings should elevate the index of suspicion [12]. Epidemiologically, neuroinvasive diseases are also less common in young and middle-aged adults [13]. The main diagnostic differential is GBS, which presents with a viral prodrome with subsequent flaccid paralysis without CSF pleocytosis. Early identification of disease allows for monitoring for complications such as respiratory failure [14], which, fortunately, our patient did not develop. 

Differentiation between a poliomyelitis-like process and other disease processes like GBS can be difficult. Paying attention to the nuances of the symptoms and test results can be very important in distinguishing between them. First, the timing of the symptoms differs with GBS, occurring weeks after an acute infection. West Nile myelitis occurs more frequently during the acute infection. Fever and leukocytosis are commonly present in West Nile myelitis, while in GBS it is not. The distribution of the paralysis also differs, with symmetrical paralysis presenting in GBS and asymmetrical paralysis and sometimes monoplegia evident in West Nile myelitis. Bowel and bladder dysregulation are also commonly seen in West Nile myelitis, while in GBS it is usually not. As previously stated, West Nile myelitis often causes encephalopathy, which is not seen in GBS. Electrodiagnostic studies in West Nile myelitis show significantly decreased or absent motor function from likely involved anterior horn cells, while GBS shows slowing or blocking of the nerve conduction from demyelination. Finally, analysis of the CSF can show both conditions having elevated protein, but West Nile myelitis can have a pleocytosis while GBS does not [10]. Albuminocytologic dissociation strongly favors GBS as the diagnosis. There have been some data regarding the use of intravenous immunoglobulin [11] and/or steroids [10], but none are well-validated in literature. Management is mainly centered on supportive care [15].

The prognosis of neurological manifestations associated with WNV infections depends primarily on the type and location of the initial lesion. It is also dependent on the severity of the illness, including encephalitis, meningitis, or myelitis. It has been shown that patients with encephalitis and meningoencephalitis demonstrate at least a 95% recovery within a year after the onset of the disease. Importantly, no statistical difference has been observed between groups [16]. However, upon discharge from the hospital, only one-third of patients suffering from encephalitis alone is reported to be fully functional. Notably, patients with meningitis alone have been shown to have a more favorable short-term prognosis [17]. In contrast, WNV-associated myelitis has been repeatedly demonstrated to have a poor functional recovery potential. In fact, most patients never fully recover. It has been suggested that only one-third of the patients experience near-complete recovery. The remaining two-thirds either have partial recovery or do not regain motor function at all. Interestingly, mental function, if impaired, is usually fully restored in patients with myelitis [18]. The degree of motor-neuron loss, absent motor responses, and absence of voluntary EMG activity are the factors known to be associated with poor prognosis [10]. Despite relatively poor prognosis in patients with myelitis, the availability of the resources aimed at improving motor recovery in patients with WNV infection (physical therapy, occupation therapy) is crucial in providing care to affected individuals. Because of these outcomes, this case demonstrates the need to have clinical suspicion for WNV in patients presenting with fevers and acute flaccid paralysis, even without any symptoms of encephalitis. 

In this case, the patient's physical therapy evaluations demonstrated significant improvement throughout his hospitalization. On the fourth day of admission, the patient had partial loss of balance and was only able to move from bed to chair with a rolling walker. The patient required moderate assistance, with at least 1-2 people needing to help him to move around. On the seventh day, the patient was able to walk 75 feet, with only one person assisting him. He continued to have very slow, but steady progress during his stay with the aid of our rehabilitation team.

## Conclusions

Though poliovirus is an entity thought to have been eradicated in the US, mimicry of poliomyelitis symptoms can still rarely occur from other viral illnesses. We presented a case of West Nile myelitis presenting with symptoms similar to poliomyelitis. This case highlights the importance of distinguishing such a motor-neuron disease from the vast spectrum of neurological diseases, and the need to have high clinical suspicion when presented with a case of fever with acute neurological signs or symptoms. Patients from regions with high mosquito burden would also have an increased risk as well. Recognition of these cases and their link to viral illnesses may decrease inappropriate diagnostic imaging and/or treatment. This case demonstrates the need for doctors to have clinical suspicion for WNV in patients presenting with fever and acute flaccid paralysis even without symptoms of encephalitis. 

## References

[1] Frauenthal HW, Manning JV (1914). Manual of infantile paralysis, with modern methods of treatment. Philadelphia Davis.

[2] (2019). Immunology and vaccine-preventable diseases. Prevention.

[3] (2019). Centers for Disease Control and Prevention: polio eradication. Prevention.

[4] Gear JH (1984). Nonpolio causes of polio-like paralytic syndromes. Rev Infect Dis.

[5] Melnick JL (1984). Enterovirus type 71 infections: a varied clinical pattern sometimes mimicking paralytic poliomyelitis. Rev Infect Dis.

[6] Akiyama N (2000). Herpes zoster infection complicated by motor paralysis. J Dermatol.

[7] Chopra JS, Banerjee AK, Murthy JM, Pal SR (1980). Paralytic rabies: a clinico-pathological study. Brain.

[8] World Health Organization (2019). World Health Organization West Nile virus. https://www.who.int/news-room/fact-sheets/detail/west-nile-virus.

[9] Kramer LD, Li J, Shi PY (2007). West Nile virus. Lancet Neurol.

[10] Leis AA, Stokic DS (2019). Neuromuscular manifestations of West Nile virus infection. Front Neurol.

[11] Ohry A, Karpin H, Yoeli D, Lazari A, Lerman Y (2001). West Nile virus myelitis. Spinal Cord.

[12] Jeha LE, Sila CA, Lederman RJ, Prayson RA, Isada CM, Gordon SM (2003). West Nile virus infection: a new acute paralytic illness. Neurology.

[13] Weinberger M, Pitlik SD, Gandacu D (2001). West Nile fever outbreak, Israel, 2000: epidemiologic aspects. Emerg Infec Dis.

[14] Sejvar JJ, Bode AV, Marfom AA (2005). West Nile virus-associated flaccid paralysis. Emergy Infect Dis.

[15] Levin SN, Lyons JL (2018). Infections of the nervous system. Am J Med.

[16] Loeb M, Hanna S, Nicolle L (2008). Prognosis after West Nile virus Infection. Ann Intern Med.

[17] Lara Jehi, Cathy A (2019). Neurologic complications of West Nile virus. http://www.clevelandclinicmeded.com/medicalpubs/diseasemanagement/neurology/neurologic-complications-west-nile-virus/#top.

[18] Johnstone J, Hanna SE, Nicolle LE, Drebot MA, Neupane B, Mahony JB, Loeb MB (2019). Prognosis of West Nile virus associated acute flaccid paralysis: a case series. J Med Case Rep.

